# 7^th^ Brazilian Guideline of Arterial Hypertension: Chapter 6 - Non-pharmacological treatment

**DOI:** 10.5935/abc.20160156

**Published:** 2016-09

**Authors:** MVB Malachias, RJS Franco, CLM Forjaz, AMG Pierin, MMG Gowdak, MRST Klein, V Matsudo

## Introduction

Non-pharmacological treatment (NPT) of AH involves body weight control, nutritional measures, practice of physical activities, smoking cessation and stress control. This chapter approaches the effects and recommendations of such measures.

### Body weight

The increase in body weight is directly related to BP increase in adults^1^ and children.^2^ The relationship between overweight and BP changes can be observed from as early as 8 years of age.^2^ In addition, the increase in visceral fat is considered a risk factor for AH.^2,3^ Reductions in body weight and AC correlate with BP reductions and metabolic improvement (Table 1).^4^ (GR: I; LE: A).

**Table 1:** Changes in body weight and in dietary ingestion and their effects on BP

Measure	Approximate SBP/DBP reduction	Recommendation
Body weight control	20%-30% BP decrease for every 5% of weight loss^1^	Maintain BMI < 25 kg/m^2^ up to 65 years of age. Maintain BMI < 27 kg/m^2^ after the age of 65 years. Maintain AC < 80 cm in women and < 94 cm in men
Dietary pattern	Reduction of 6.7/3.5 mm Hg^35^	Adopt DASH diet
Sodium intake restriction	Reduction of 2-7 mm Hg in SBP and 1-3 mm Hg in DBP with progressive reduction of 2.4-1.5 g of sodium/day, respectively^12^	Limit daily sodium intake to 2.0 g (5 g of sodium chloride)
Moderation in alcohol intake	Reduction of 3.31/2.04 mm Hg with the reduction from 3-6 to 1-2 doses/day^34^	Limit daily alcohol intake to 1 dose for women and low-weight individuals, and 2 doses for men

BMI: body mass index; AC: abdominal circumference; SBP: systolic blood pressure; DBP: diastolic blood pressure.

* One dose contains 14g of ethanol, and is equivalent to 350 mL of beer, 150 mL of wine, and 45 mL of distilled beverage.^36^

### Nutritional aspects

#### Dietary pattern

The success of AH treatment with nutritional measures depends on the adoption of a healthy and sustainable dietary plan.^5^ The use of radical diets results in treatment dropout.^6^ The focus on one single nutrient or food has given space to the total dietary pattern analysis, which allows assessing the synergism between nutrients/foods.^7^

The DASH (*Dietary Approaches to Stop Hypertension*) diet emphasizes the consumption of fruits, vegetables and low-fat dairy products, includes the ingestion of whole cereals, chicken, fish and nuts, and recommends a reduction in the ingestion of red meat, candies and sugary beverages. That diet is rich in potassium, calcium, magnesium and fibers, and contains reduced amounts of cholesterol and of total and saturated fat. Adopting that dietary pattern reduces BP.^8,9^ (GR: I; LE: A).

The Mediterranean diet is rich in fruits, vegetables and whole cereals, but has large amounts of olive oil (source of monounsaturated fats) and includes the consumption of fish and nuts, in addition to the moderate ingestion of wine.^7^ Despite the limited number of studies, the adoption of the Mediterranean diet seems to low BP.^10^ (GR: IIa; LE: B).

Vegetarian diets recommend the consumption of foods of plant origin, specially fruits, vegetables, grains and pulses. They exclude or rarely include meats, and some include dairy products, eggs and fish. They have been associated with lower BP levels.^11^ (GR: IIa; LE: B).

#### Reduction in sodium intake

The increase in sodium intake is related to BP elevation.^12^ However, the impact of sodium intake on CV health is controversial. Some studies have suggested that very low sodium intake increases the risk for CVD, while others argue that the decrease in sodium intake decreases the CV risk,^13^ and that benefit is even higher with marked restriction of sodium intake.^14^

Limiting daily sodium intake to 2.0 g is associated with BP reduction.^15^ The Brazilian mean sodium intake is 11.4 g/day.^16^ (GR: IIa; LE: B).

#### Unsaturated fatty acids

Omega-3 fatty acids originating from fish oils (eicosapentaenoic and docosahexaenoic acids, EPA and DHA, respectively) are associated with a mild reduction in BP. Recent studies have indicated that the EPA+DHA ingestion ≥ 2 g/day reduces BP, and lower doses (1-2 g/day) reduce only SBP.^17^ (GR: IIa; LE: B).

In addition, the consumption of monounsaturated fatty acids has been associated with BP reduction.^18^ (GR: IIb; LE: B).

#### Fibers

Soluble fibers are present in oat bran, pectin (fruits) and starch (oat, barley and pulses: beans, chickpeas, lentils and green peas), while insoluble fibers are present in cellulose (wheat), hemicellulose (whole grains) and lignin (vegetables). The ingestion of fibers, mainly beta-glucan originating from oat and barley, causes a mild decrease in BP.^19^ (GR: IIb; LE: B).

#### Nuts

The consumption of nuts helps control several CVRF, but few studies have related that consumption to BP reduction.^20^ A meta-analysis has concluded that the ingestion of different types of nuts could reduce BP.^21^ (GR: IIb; LE: B).

#### Dairy products and vitamin D

There is evidence that the ingestion of dairy products, especially low-fat ones, reduces BP.^22^ Milk contains several components, such as calcium, potassium and bioactive peptides, that can decrease BP.^23^ (GR: IIb; LE: B).

Some studies have shown that low serum levels of vitamin D are associated with a greater incidence of AH.^24^ However, studies on vitamin D supplementation have failed to show BP reduction.^25^ (GR: III; LE: B).

#### Garlic

Garlic has innumerous bioactive components, such as allicin (found in raw garlic) and s-allyl cysteine (found in processed garlic). Mild BP decrease has been reported with supplementation with several forms of garlic.^26,27^ (GR: IIb; LE: B).

#### Coffee and green tea

Coffee, although rich in caffeine, substance with an acute pressor effect, has polyphenols that can favor BP reduction.^28^ Recent studies have suggested that coffee intake at usual doses is associated with neither higher AH incidence nor BP elevation.^29^ Coffee intake should not exceed low to moderate amounts. (GR: IIa; LE: B).

In addition to being rich in polyphenols, especially catechins, green tea has caffeine. There is no consensus, but some studies have suggested that green tea might reduce BP when consumed at low doses, because greater doses have a higher caffeine content and can increase BP.^30^ Green tea consumption is recommended at low doses. (GR: IIb; LE: B).

#### Bitter chocolate

Chocolate at least 70% cacao can cause mild BP reduction, because of its high polyphenol content.^31^ (GR: IIb; LE: B).

#### Alcohol

Usual alcohol consumption increases BP linearly, and its excessive consumption associates with an increase in the AH incidence.^32,33^ A 10-g/day increment in alcohol ingestion is estimated to increase BP by 1 mm Hg,^32^ and a decrease in that consumption reduces BP.^3^ Moderation in alcohol intake is recommended. (GR: I; LE: B).

### Physical activity/physical exercise

Physical activity refers to any body movement that increases energy expenditure, such as street walking, stair climbing, domestic chores, and recreational activities. The term 'physical exercise' refers to planned, structured, repetitive and purposeful physical activity. In addition, sedentary lifestyle, measured by the time spent sitting, has CV health implications (Tables 2 and 3).

**Table 2:** Evidence of physical activity and physical exercise for BP reduction

Measure	Approximate SBP/DBP reduction
Daily physical activity	3.6/5.4 mm Hg^42^
Aerobic exercise	2.1/1.7 in prehypertensives8.3/5.2 mm Hg in hypertensives^43^
Dynamic resistance exercise	4.0/3.8 mm Hg in prehypertensivesNo reduction in hypertensives^43^

**Table 3:** Recommendations regarding physical activity and physical exercise

**For all hypertensives - Population recommendation – Physical activity practice**
Moderate, continuous (1 x 30 min) or cumulative (2 x 15 min or 3 x 10 min) physical activity: at least 30 min/day, 5 - 7 days/week.
**For greater benefits - Individual recommendation - Physical exercise **
Aerobic training complemented with resistance training
Aerobic training
Several modalities: walking, running, dancing, swimming.
At least 3 times/week. Ideally: 5 times/week.
Minimum of 30 min. Ideally: 40 - 50 min.
Moderate intensity defined as:
1) Higher intensity, but still being able to talk (without being breathless)
2) Feeling mildly to moderately tired
3) Maintain training HR calculated as follows: Training HR = (maximum HR – resting HR) x % + resting HR where:maximum HR: obtained either on a maximum exercise test, using the regular medications, or by calculating maximum HR estimated according to age (220 - age). The formula cannot be applied to hypertensives with heart disease or on beta-blockers or nondihydropyridine calcium channel blockers. Resting HR: measured after 5-minute resting lying down. %: use 50% as lower threshold, and 70% as upper threshold.
**Resistance training**
2 - 3 times/week.
8 - 10 exercises for the large muscle groups, prioritizing unilateral execution, when possible.
1 - 3 sets
10 - 15 repetitions up to moderate fatigue (reduction in the velocity of movement and tendency towards apnea)
Long passive pauses - 90 - 120 s

#### Physical inactivity/activity

Physical inactivity is "a major public health problem",^37^ because it is the most prevalent RF and the second cause of death worldwide.^38^ Survival is shorter among individuals who spend most of their time sitting than among those who do not.^39^ There is a direct relationship between the time spent sitting or watching TV and BP.^40^ To reduce the sitting time and to stand up for at least 5 minutes for every 30 minutes sited are recommended. (GR: IIb; LE: B).

Regular physical activity can benefit both AH prevention and treatment, and reduces CV morbidity and mortality. Active individuals have a 30% lower risk of developing AH as compared to those with a sedentary lifestyle.^41^ The increase in daily physical activity reduces BP.^42^ Physical activity practice should be encouraged for the entire population, and no previous test is required. The individual should be instructed to seek a doctor if any discomfort occurs during the physical activity practice. (GR: I; LE: A).

#### Physical exercise

The AH treatment can derive additional benefits from structured physical exercise practice, characterizing a customized training.

#### Aerobic exercise

Aerobic training reduces casual BP of prehypertensive and hypertensive individuals.^43^ In addition, it reduces BP during wakefulness for hypertensives^44^ and lowers BP in situations of physical, mental and psychological stress.^45^ Aerobic training is recommended as the preferential exercise type for AH prevention and treatment. (GR: I; LE: A).

#### Dynamic and static resistance exercise

Dynamic or isotonic resistance training (contraction of localized body segments with joint movement) reduces BP of prehypertensive individuals, but has no effect in hypertensives. However, there are only four randomized, controlled studies on that exercise type for AH.^43^ Static or isometric resistance training (contraction of localized body segments without joint movement) reduces BP of hypertensives, but the studies have used small muscle masses, thus, further information is required prior to its recommendation.^46^ Dynamic resistance training is recommended to complement aerobic training for AH. (GR: IIa; LE: B).

#### Caution

Hypertensives with higher BP levels or with more than three RF, DM, TOD or heart disease should undergo exercise testing before engaging in moderate-intensity physical exercises. In addition, every hypertensive engaging in competitive sports or high-performance exercise should undergo complete CV assessment.^45^ (GR: IIa; LE: C).

### Smoking cessation

Smoking increases the risk for more than 25 diseases, including CVD.^47,48^ The smoking habit hinders AH control,^49^ knowledge about SAH^50^ and adherence to antihypertensive medications.^51^ However, there is no evidence that smoking cessation reduces BP. (GR: III, LE: B).

### Slow breathing

Slow or guided breathing requires respiratory rate reduction to 6-10 breaths/minute for 15-20 minutes/day to promote casual BP reduction (SBP: -3.67; 95% confidence interval: -5.99 to -1.39; and DBP: -2.51; 95% confidence interval: -4.15 to 0.87) after 8 weeks of treatment.^52^ (GR: IIa; LE: B).

### Stress control

Studies on stress management techniques emphasize the importance of behavioral psychotherapies and meditation,^53-55^ biofeedback and relaxation^56^ practices in AH treatment. Despite methodological contradictions, clinical indications have revealed a strong trend towards BP reduction when those techniques are performed separately or combined.^56^ (GR: IIa; LE: B).

### Multiprofessional team

The multiprofessional approach is mainly aimed at AH control, which is not satisfactory in our setting. Epidemiological studies have shown a 10% to 57.6% variation^57^ in that control. The multiprofessional team promotes better AH control,^58^ which is directly related to adherence to pharmacological and non-pharmacological treatment. The multiprofessional team can consist of all professionals managing hypertensive patients: doctors, nurses, technicians and nurse aides, nutritionists, psychologists, social workers, physical therapists, physical education coaches, music therapists, chemists, educators, media professionals, administrative workers and community health agents.

## References

[1] DeMarco VG, Aroor AR, Sowers JR (2014). The pathophysiology of hypertension in patients with
obesity. Nat Rev Endocrinol.

[2] Vaneckova I, Maletinska L, Behuliak M, Nagelova V, Zicha J, Kunes J (2014). Obesity-related hypertension: possible pathophysiological
mechanisms. J Endocrinol.

[3] Fuentes E, Fuentes F, Vilahur G, Badimon L, Palomo I (2013). Mechanisms of chronic state of inflammation as mediators that
link obese adipose tissue and metabolic syndrome. Mediators Inflamm.

[4] Guimaraes IC, de Almeida AM, Santos AS, Barbosa DB, Guimaraes AC (2008). Blood pressure: effect of body mass index and of waist
circumference on adolescents. Arq Bras Cardiol.

[5] Greenberg I, Stampfer MJ, Schwarzfuchs D, Shai I, Group D (2009). Adherence and success in long-term weight loss diets: the dietary
intervention randomized controlled trial (DIRECT). J Am Coll Nutr.

[6] Alhassan S, Kim S, Bersamin A, King AC, Gardner CD (2008). Dietary adherence and weight loss success among overweight women:
results from the A TO Z weight loss study. Int J Obes (Lond).

[7] Martinez-Gonzalez MA, Bes-Rastrollo M (2014). Dietary patterns, mediterranean diet, and cardiovascular
disease. Curr Opin Lipidol.

[8] Appel LJ, Moore TJ, Obarzanek E, Vollmer WM, Svetkey LP, Sacks FM, DASH Collaborative Research Group (1997). A clinical trial of the effects of dietary patterns on blood
pressure. N Engl J Med.

[9] Sacks FM, Svetkey LP, Vollmer WM, Appel LJ, Bray GA, Harsha D, DASH-Sodium Collaborative Research Group (2001). Effects on blood pressure of reduced dietary sodium and the
Dietary Approaches to Stop Hypertension (DASH) diet. N Engl J Med.

[10] Domenech M, Roman P, Lapetra J, Garcia de la Corte FJ, Sala-Vila A, de la Torre R (2014). Mediterranean diet reduces 24-hour ambulatory blood pressure,
blood glucose, and lipids: one-year randomized, clinical
trial. Hypertension.

[11] Yokoyama Y, Nishimura K, Barnard ND, Takegami M, Watanabe M, Sekikawa A (2014). Vegetarian diets and blood pressure: a
meta-analysis. JAMA Intern Med.

[12] Eckel RH, Jakicic JM, Ard JD, de Jesus JM, Miller NH, Hubbard VS (2014). 2013 AHA/ACC Guideline on Lifestyle Management to Reduce
Cardiovascular Risk A Report of the American College of Cardiology/American
Heart Association Task Force on Practice Guidelines. Journal of the American College of Cardiology.

[13] Graudal N, Jurgens G, Baslund B, Alderman MH (2014). Compared with usual sodium intake, low- and excessive-sodium
diets are associated with increased mortality: a
meta-analysis. Am J Hypertens.

[14] He FJ, Li J, Macgregor GA (2013). Effect of longer term modest salt reduction on blood pressure:
Cochrane systematic review and meta-analysis of randomised
trials. BMJ.

[15] O'Donnell M, Ment A, Rangarajan S, McQueen MJ, Wang X, Liu L (2014). Urinary sodium and potassium excretion, mortality, and
cardiovascular events. N Engl J Med.

[16] Instituto Brasileiro de Geografia e Estatística (2011). Pesquisa de orçamentos familiares 2008-2009: análise do
consumo alimentar pessoal no Brasil.

[17] Miller PE, Van Elswyk M, Alexander DD (2014). Long-chain omega-3 fatty acids eicosapentaenoic acid and
docosahexaenoic acid and blood pressure: a meta-analysis of randomized
controlled trials. Am J Hypertens.

[18] Schwingshackl L, Strasser B, Hoffmann G (2011). Effects of monounsaturated fatty acids on cardiovascular risk
factors: a systematic review and meta-analysis. Ann Nutr Metab.

[19] Evans CE, Greenwood DC, Threapleton DE, Cleghorn CL, Nykjaer C, Woodhead CE (2015). Effects of dietary fibre type on blood pressure: a systematic
review and meta-analysis of randomized controlled trials of healthy
individuals. J Hypertens.

[20] Salas-Salvado J, Guasch-Ferre M, Bullo M, Sabate J (2014). Nuts in the prevention and treatment of metabolic
syndrome. Am J Clin Nutr.

[21] Mohammadifard N, Salehi-Abargouei A, Salas-Salvado J, Guasch-Ferre M, Humphries K, Sarrafzadegan N (2015). The effect of tree nut, peanut, and soy nut consumption on blood
pressure: a systematic review and meta-analysis of randomized controlled
clinical trials. Am J Clin Nutr.

[22] Park KM, Cifelli CJ (2013). Dairy and blood pressure: a fresh look at the
evidence. Nutr Rev.

[23] Fekete AA, Givens DI, Lovegrove JA (2013). The impact of milk proteins and peptides on blood pressure and
vascular function: a review of evidence from human intervention
studies. Nutr Res Rev.

[24] Kunutsor SK, Apekey TA, Steur M (2013). Vitamin D and risk of future hypertension: meta-analysis of
283,537 participants. Eur J Epidemiol.

[25] Beveridge LA, Struthers AD, Khan F, Jorde R, Scragg R, Macdonald HM (2015). Effect of vitamin D supplementation on blood pressure: a
systematic review and meta-analysis incorporating individual patient
data. JAMA Intern Med.

[26] Ried K, Fakler P (2014). Potential of garlic (Allium sativum) in lowering high blood
pressure: mechanisms of action and clinical relevance. Integr Blood Press Control.

[27] Rohner A, Ried K, Sobenin IA, Bucher HC, Nordmann AJ (2015). A systematic review and metaanalysis on the effects of garlic
preparations on blood pressure in individuals with
hypertension. Am J Hypertens.

[28] Rebello SA, van Dam RM (2013). Coffee consumption and cardiovascular health: getting to the
heart of the matter. Curr Cardiol Rep.

[29] Mesas AE, Leon-Munoz LM, Rodriguez-Artalejo F, Lopez-Garcia E (2011). The effect of coffee on blood pressure and cardiovascular disease
in hypertensive individuals: a systematic review and
meta-analysis. Am J Clin Nutr.

[30] Peng XL, Zhou R, Wang B, Yu XP, Yang XH, Liu K (2014). Effect of green tea consumption on blood pressure: A
meta-analysis of 13 randomized controlled trials. Sci Rep.

[31] Pereira T, Maldonado J, Laranjeiro M, Coutinho R, Cardoso E, Andrade I (2014). Central arterial hemodynamic effects of dark chocolate ingestion
in young healthy people: a randomized and controlled trial. Cardiol Res Pract.

[32] Fan AZ, Li Y, Elam-Evans LD, Balluz L (2013). Drinking pattern and blood pressure among non-hypertensive
current drinkers: findings from 1999-2004 National Health and Nutrition
Examination Survey. Clin Epidemiol.

[33] Taylor B, Irving HM, Baliunas D, Roerecke M, Patra J, Mohapatra S (2009). Alcohol and hypertension: gender differences in dose-response
relationships determined through systematic review and
meta-analysis. Addiction.

[34] Xin X, He J, Frontini MG, Ogden LG, Motsamai OI, Whelton PK (2001). Effects of alcohol reduction on blood pressure: a meta-analysis
of randomized controlled trials. Hypertension.

[35] Saneei P, Salehi-Abargouei A, Esmaillzadeh A, Azadbakht L (2014). Influence of Dietary Approaches to Stop Hypertension (DASH) diet
on blood pressure: a systematic review and meta-analysis on randomized
controlled trials. Nutr Metab Cardiovasc Dis.

[36] O'Keefe JH, Bhatti SK, Bajwa A, DiNicolantonio JJ, Lavie CJ (2014). Alcohol and cardiovascular health: the dose makes the poison...or
the remedy. Mayo Clin Proc.

[37] Blair SN (2009). Physical inactivity: the biggest public health problem of the
21st century. Br J Sports Med.

[38] Lee IM, Shiroma EJ, Lobelo F, Puska P, Blair SN, Katzmarzyk PT (2012). Effect of physical inactivity on major non-communicable diseases
worldwide: an analysis of burden of disease and life
expectancy. Lancet.

[39] Katzmarzyk PT, Church TS, Craig CL, Bouchard C (2009). Sitting time and mortality from all causes, cardiovascular
disease, and cancer. Med Sci Sports Exerc.

[40] Thorp AA, Healy GN, Owen N, Salmon J, Ball K, Shaw JE (2010). Deleterious associations of sitting time and television viewing
time with cardiometabolic risk biomarkers: Australian Diabetes, Obesity and
Lifestyle (AusDiab) study 2004-2005. Diabetes Care.

[41] Fagard RH (2005). Physical activity, physical fitness and the incidence of
hypertension. J Hypertens.

[42] Dunn AL, Marcus BH, Kampert JB, Garcia ME, Kohl 3rd HW, Blair SN (1999). Comparison of lifestyle and structured interventions to increase
physical activity and cardiorespiratory fitness: a randomized
trial. JAMA.

[43] Cornelissen VA, Smart NA (2013). Exercise training for blood pressure:a systematic review and
meta-analysis. J Am Heart Assoc.

[44] Cornelissen VA, Buys R, Smart NA (2013). Endurance exercise beneficially affects ambulatory blood
pressure: a systematic review and meta-analysis. J Hypertens.

[45] Fagard RH (2011). Exercise therapy in hypertensive cardiovascular
disease. Prog Cardiovasc Dis.

[46] Carlson DJ, Dieberg G, Hess NC, Millar PJ, Smart NA (2014). Isometric exercise training for blood pressure management: a
systematic review and meta-analysis. Mayo Clin Proc.

[47] Nobre F, Ribeiro AB, Mion Jr D (2010). Control of arterial pressure in patients undergoing
anti-hypertensive treatment in Brazil: Controlar Brazil. Arq Bras Cardiol.

[48] Yun M, Li S, Sun D, Ge S, Lai CC, Fernandez C (2015). Tobacco smoking strengthens the association of elevated blood
pressure with arterial stiffness: the Bogalusa Heart Study. J Hypertens.

[49] De Giusti M, Dito E, Pagliaro B, Burocchi S, Laurino FI, Tocci G (2012). A survey on blood pressure levels and hypertension control in a
sample of the Italian general population. High Blood Press Cardiovasc Prev.

[50] Guessous I, Bochud M, Theler JM, Gaspoz JM, Pechere-Bertschi A (2012). 1999-2009 Trends in prevalence, unawareness, treatment and
control of hypertension in Geneva, Switzerland. PLoS One.

[51] Mion Jr D, Pierin AM, Bensenor IM, Marin JC, Costa KR, Henrique LF (2010). Hypertension in the city of Sao Paulo: self-reported prevalence
assessed by telephone surveys. Arq Bras Cardiol.

[52] Mahtani KR, Nunan D, Heneghan CJ (2012). Device-guided breathing exercises in the control of human blood
pressure: systematic review and meta-analysis. J Hypertens.

[53] Bai Z, Chang J, Chen C, Li P, Yang K, Chi I (2015). Investigating the effect of transcendental meditation on blood
pressure: a systematic review and meta-analysis. J Hum Hypertens.

[54] Campbell TS, Labelle LE, Bacon SL, Faris P, Carlson LE (2012). Impact of Mindfulness-Based Stress Reduction (MBSR) on attention,
rumination and resting blood pressure in women with cancer: a
waitlist-controlled study. J Behav Med.

[55] Sharma M, Rush SE (2014). Mindfulness-based stress reduction as a stress management
intervention for healthy individuals: a systematic review. J Evid Based Complementary Altern Med.

[56] Brook RD, Appel LJ, Rubenfire M, Ogedegbe G, Bisognano JD, Elliott WJ (2013). Beyond medications and diet: alternative approaches to lowering
blood pressure: a scientific statement from the american heart
association. Hypertension.

[57] Pinho Nde A, Pierin AM (2013). Hypertension control in brazilian publications. Arq Bras Cardiol.

[58] Glynn LG, Murphy AW, Smith SM, Schroeder K, Fahey T (2010). Interventions used to improve control of blood pressure in
patients with hypertension. Cochrane Database Syst Rev.

